# NF-κB-regulated microRNA-574-5p underlies synaptic and cognitive impairment in response to atmospheric PM_2.5_ aspiration

**DOI:** 10.1186/s12989-017-0215-3

**Published:** 2017-08-29

**Authors:** Tingting Ku, Ben Li, Rui Gao, Yingying Zhang, Wei Yan, Xiaotong Ji, Guangke Li, Nan Sang

**Affiliations:** College of Environment and Resource, Research Center of Environment and Health, Shanxi University, Taiyuan, Shanxi 030006 China

**Keywords:** PM_2.5_, β-site amyloid precursor protein cleaving enzyme 1 (β-secretase, BACE1), MicroRNA-574-5p (miR-574-5p), NF-κB, Synaptic dysfunction, Spatial memory and learning

## Abstract

**Background:**

PM_2.5_ (particulate matter ≤ 2.5 μm) is one of the leading environmental risk factors for the global burden of disease. Whereas increasing evidence has linked the adverse roles of PM_2.5_ with cardiovascular and respiratory diseases, limited but growing emerging evidence suggests that PM_2.5_ exposure can affect the nervous system, causing neuroinflammation, synaptic dysfunction and cognitive deterioration. However, the molecular mechanisms underlying the synaptic and cognitive deficits elicited by PM_2.5_ exposure are largely unknown.

**Methods:**

C57BL/6 mice received oropharyngeal aspiration of PM_2.5_ (1 and 5 mg/kg bw) every other day for 4 weeks. The mice were also stereotaxically injected with β-site amyloid precursor protein cleaving enzyme 1 (β-secretase, BACE1) shRNA or LV-miR-574-5p lentiviral constructs in the absence or presence of PM_2.5_ aspiration at 5 mg/kg bw every other day for 4 weeks. Spatial learning and memory were assessed with the Morris water maze test, and synaptic function integrity was evaluated with electrophysiological recordings of long-term potentiation (LTP) and immunoblot analyses of glutamate receptor subunit expression. The expression of α-secretase (ADAM10), BACE1, and γ-secretase (nicastrin) and the synthesis and accumulation of amyloid β (Aβ) were measured by immunoblot and enzyme-linked immunosorbent assay (ELISA). MicroRNA (miRNA) expression was screened with a microRNA microarray analysis and confirmed by real-time quantitative reverse transcription PCR (qRT-PCR) analysis. Dual-luciferase reporter gene and chromatin immunoprecipitation (ChIP) analyses were used to detect the binding of miR-574-5p in the 3’UTR of BACE1 and NF-κB p65 in the promoter of miR-574-5p, respectively.

**Results:**

PM_2.5_ aspiration caused neuroinflammation and deteriorated synaptic function integrity and spatial learning and memory, and the effects were associated with the induction of BACE1. The action was mediated by NF-κB p65-regulated downregulation of miR-574-5p, which targets BACE1. Overexpression of miR-574-5p in the hippocampal region decreased BACE1 expression, restored synaptic function, and improved spatial memory and learning following PM_2.5_ exposure.

**Conclusions:**

Taken together, our findings reveal a novel molecular mechanism underlying impaired synaptic and cognitive function following exposure to PM_2.5_, suggesting that miR-574-5p is a potential intervention target for the prevention and treatment of PM_2.5_-induced neurological disorders.

**Electronic supplementary material:**

The online version of this article (10.1186/s12989-017-0215-3) contains supplementary material, which is available to authorized users.

## Background

Air pollution contributes to a broad array of acute and chronic health effects, with an estimated impact of 5.5 million deaths per year worldwide [1]. Air pollution represents a diverse mixture of substances, including particulate matter (PM), gases, organic compounds, and toxic metals [2]. Among these, fine particles (PM ≤ 2.5 μm; PM_2.5_) appear to be one of the most harmful components and greatest health threats and have been heavily implicated in disease [3]. While increasing evidence has linked the adverse effects of PM_2.5_ to cardiovascular and respiratory diseases, recent limited but growing evidence suggests that PM_2.5_ exposure can affect the nervous system, indicating an association between exposure to particulate air pollution and cognitive function [4–9]. This supposition is supported by animal and human studies showing that air pollution induces neuroinflammation and neurodegeneration and alters cognitive function [10–19]. Changes in cognitive function are the main performance factors resulting from nervous system dysfunction [20] and are the prodromal symptom of many neurological diseases [21, 22]. Synaptic plasticity is considered to be the biological basis of cognitive activity at the cellular level [23]. Glutamate receptors, including *N*-methyl-D-aspartate (NMDA) receptors and α-amino-3-hydroxy-5-methyl-4-isoxazolepropionic acid (AMPA) receptors, are responsible for the functional integrity of excitatory synapses and are central to the molecular mechanisms of learning and memory [24]. Individuals displaying inflammation and neurodegeneration resulting from air pollutants may be more likely to develop cognitive deficits [25] and may be at greater risk for the development of progressive neurodegenerative diseases such as Alzheimer’s disease (AD) [26]. Importantly, recent experimental studies have demonstrated that PM_2.5_ alters synaptic gene expression, causes synaptic dysfunction, and promotes the development of early AD-like neuropathology [16, 27, 28]. However, the molecular mechanisms underlying the synaptic and cognitive deficits elicited by PM_2.5_ exposure are largely unknown.

MicroRNAs (miRNAs), a group of 18- to 25-nucleotide-long noncoding RNA molecules, are increasingly being recognized as key regulators that target proteins at the translational level [29]. Importantly, altered miRNA profiles are associated with cognitive deterioration and neurodegenerative diseases, including AD [30–32]. miRNA loss in mice due to deficient expression of Dicer or DGCR8, two essential components of the miRNA biogenesis pathway [33], leads to alterations in synaptic protein expression, dendritic spines, synaptic transmission, and learning and memory [34, 35]. Although a few studies have demonstrated that miRNA dysregulation is associated with exposure to PM, diesel exhaust particles and carbon black nanoparticles [36–39], little is known about whether PM_2.5_ exposure alters the miRNA profile in the brain and whether and how the dysregulated miRNAs mediate PM exposure-induced synaptic and cognitive deficits.

β-Site amyloid precursor protein (APP) cleaving enzyme 1 (β-secretase, BACE1) is a critical enzyme catalyzing amyloid β (Aβ) peptide generation by cleaving APP, and BACE1 activation is a hallmark of early stage cognitive deficits and plays an important role in progressive conversion to AD. Previously, several miRNAs have been demonstrated to be involved in post-transcriptional regulation of BACE1, including miR-9, miR-29, miR-107, miR-186, miR-188, miR-298, and miR-328 [40, 41]. Alterations in specific miRNA levels can upregulate expression and stability of BACE1 protein, which in turn contributes to synaptic and cognitive deficits. In the present study, we show that PM_2.5_ aspiration impaired synaptic and cognitive function, and the effects were associated with the induction of BACE1. Furthermore, we provide evidence that this action was mediated by NF-κB p65-induced downregulation of miR-574-5p, which targets BACE1. Importantly, overexpression of miR-574-5p in the hippocampal region decreased BACE1 expression, restored synaptic function, and improved spatial memory and learning following PM_2.5_ aspiration. Our results reveal a novel miRNA-modulated mechanism responsible for synaptic and cognitive impairments caused by PM_2.5_ exposure.

## Results

During the experiment, none of the animals died, and food intake and body weight remained unchanged following PM_2.5_ aspiration.

### PM_2.5_ aspiration induces lung and systemic inflammation

**Fig. 1:**
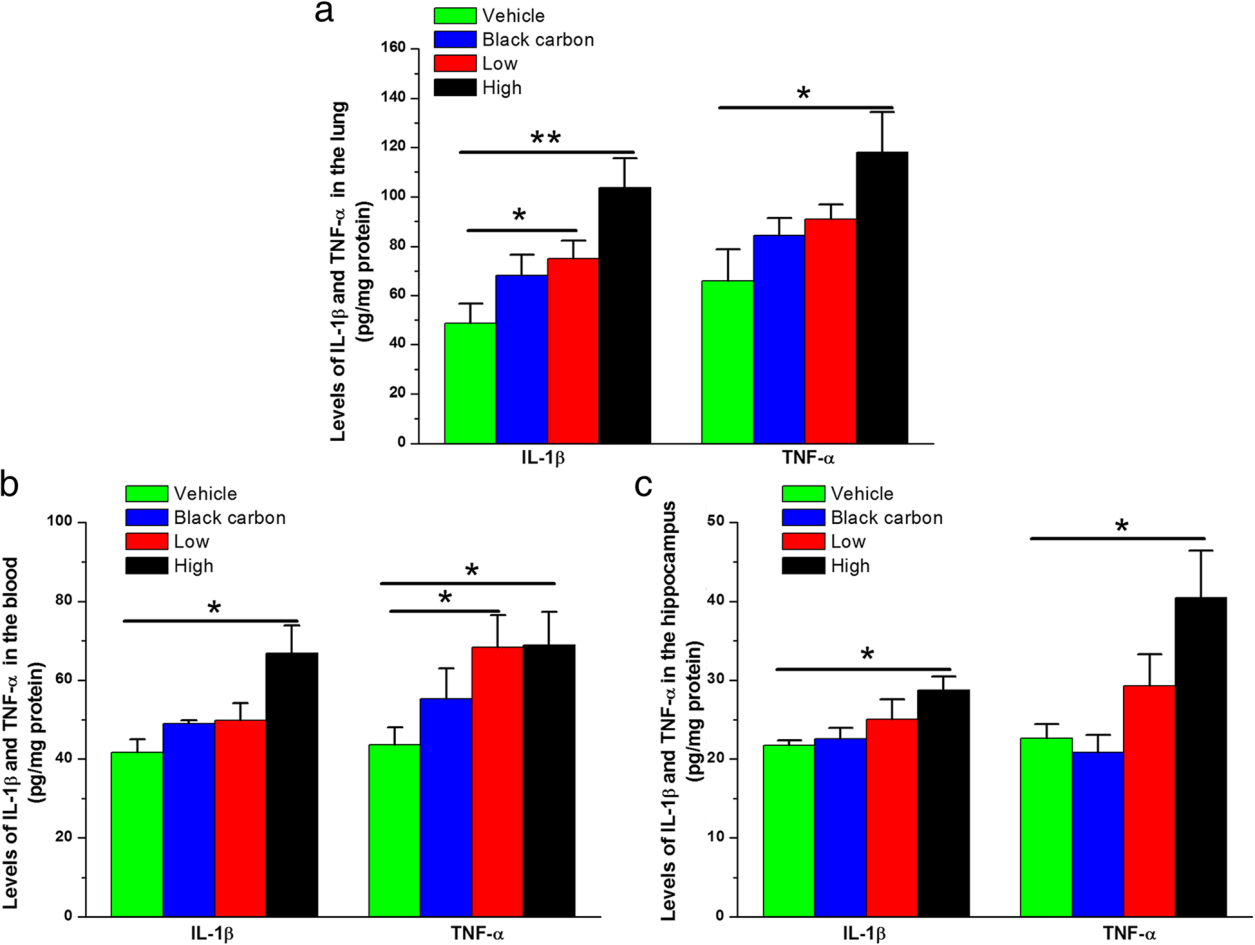
PM_2.5_ aspiration increases IL-1β and TNF-α levels in the (**a**) lung, (**b**) blood and (**c**) hippocampus. The data are expressed as the means ± SE (*n* = 6 mice/group). *P < 0.05 and **P < 0.01. Low = 1 mg/kg PM_2.5_; High = 5 mg/kg PM_2.5_

Neuroinflammation is one of the main pathogenic mechanisms leading to synaptic and cognitive deficits, and PM can invade the brain through inflammatory mediators released from primary entry organs or secondary deposition sites [42]. To clarify this possibility, we first detected the lung and systemic inflammation response to PM_2.5_ and an inert particle (black carbon) at the same level of exposure. As shown in Fig. 1, PM_2.5_ aspiration dose-dependently increased IL-1β and TNF-α levels in the lungs, blood and brain, and significant changes were observed at 1 mg/kg bw for IL-1β in the lungs and TNF-α in the blood and at 5 mg/kg bw for both IL-1β and TNF-α in the lungs, blood and brain. The results suggest the possibility that PM_2.5_ exposure affects the nervous system through systemic inflammation.

### PM_2.5_ exposure stimulates BACE1 expression and deteriorates functional synaptic integrity and spatial learning and memory

**Fig. 2:**
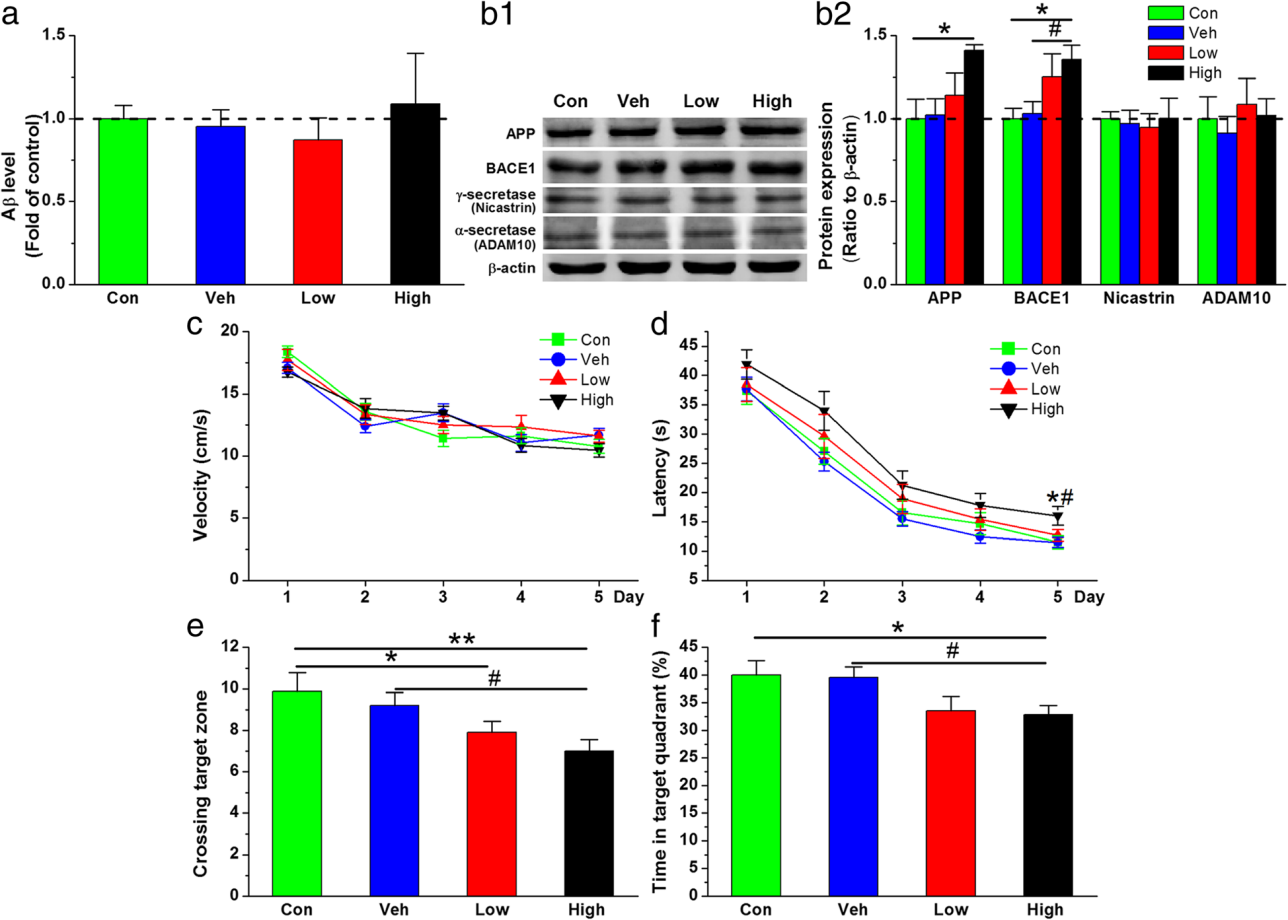
PM_2.5_ exposure stimulates BACE1 expression and impairs spatial learning and memory. **a** ELISA detection of Aβ42 content. (**b1**) Representative protein bands of immunoblot analysis for APP, BACE1, *γ*-secretase (nicastrin) and *α*-secretase (ADAM10). (**b2**) Quantification of APP, BACE1, *γ*-secretase (nicastrin) and *α*-secretase (ADAM10) expression. The data are presented as the means ± SE (*n* = 4 to 8 mice/group). **c** Swim velocity over 5 days of invisible training to find the hidden platform in the Morris water maze. **d** Learning curve over 5 days of invisible training to find the hidden platform in the Morris water maze. Retention memory was determined using a probe trial test conducted 24 h after 5 days of training. During the probe test, the platform was removed from the pool. **e** Number of times crossing the target zone. **f** Percentage of time spent in the target quadrant. The data are expressed as the means ± SE (*n* = 13 to 14 mice/group). */^#^ *P* < 0.05; **P < 0.01. Con = control group. Veh = vehicle control; Low = 1 mg/kg PM_2.5_; High = 5 mg/kg PM_2.5。_In all figures, unless stated otherwise, the *dotted line* crossing the y-axis at 1.0 represents data normalized to the control or vehicle control

PM_2.5_ has been reported to alter the brain inflammatory milieu and accelerate AD progression, mainly through BACE1-catalyzed APP cleavage [27]. Additionally, the sequential cleavage of APP by BACE1 [43, 44] and γ-secretase (a complex composed of presenilin, nicastrin, Aph1 and Pen2) to Aβ [45, 46] is central to the pathophysiology of AD and is likely to play an early role in cognitive dysfunction. Aβ formation can be prevented by the activities of α-secretase, which has been identified as ADAM10 [47]. Thus, we hypothesized that PM_2.5_ aspiration might promote BACE1- and γ-secretase- but not α-secretase-mediated cleavage of APP, leading to Aβ synthesis and accumulation and synaptic and cognitive impairment. To test this idea, we treated animals with PM_2.5_ at two dosing regimens (1 and 5 mg/kg bw) and first measured the levels of hippocampal Aβ and APP, BACE1, γ-secretase (nicastrin) and α-secretase (ADAM10) expression. Unexpectedly, PM_2.5_ aspiration did not increase production or deposition of Aβ42 (Fig. 2a). However, the expression of APP and BACE1, but not γ-secretase (nicastrin) or α-secretase (ADAM10), were elevated, and a significant difference was found following PM_2.5_ aspiration at 5 mg/kg bw (Fig. 2b).

Not only does BACE1 initiate Aβ formation, but increased BACE1 activity may affect normal synaptic functioning. Therefore, BACE1 is classified as a hallmark of early cognitive impairment [48, 49]. Deficits in learning and memory are the principal characteristic of cognitive dysfunction. Therefore, we performed the Morris water maze test to assess whether PM_2.5_ exposure impairs spatial learning and memory. During the training trials, PM_2.5_-exposed mice exhibited a longer time to reach the hidden platform than the vehicle control animals, but no alteration in swimming speed was observed (Fig. 2c and d). In the probe trial, the number of crossings through the target zone (Fig. 2e) and the time spent in the target quadrant (Fig. 2f) were decreased compared with those in the vehicle control condition, and there was a statistically significant difference between the mice treated with vehicle and those treated with PM_2.5_ at 5 mg/kg bw. The reduced number of crossings and shorter duration stay in the target quadrant demonstrated a decrease in memory retention, indicating that PM_2.5_ aspiration impaired spatial learning and memory.

**Fig. 3:**
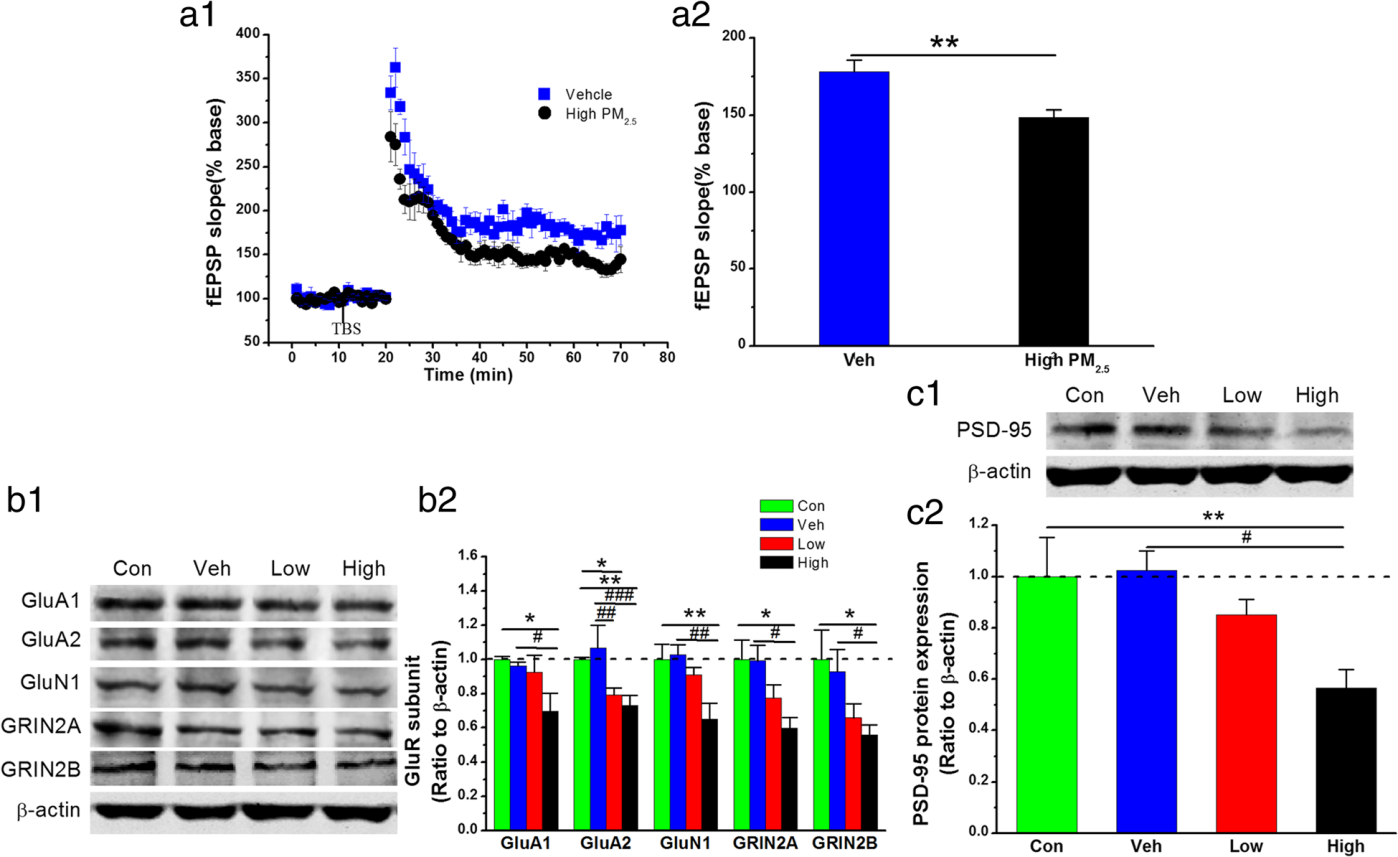
PM_2.5_ exposure impairs functional synaptic integrity. (**a1**) Time courses of changes in the field excitatory postsynaptic potential (fEPSP) slope under different treatments. (**a2**) Mean values of the fEPSP slope averaged from 56 to 60 min after theta burst stimulation (TBS). The data are expressed as the means ± SE (*n =* 6 mice/group). (**b1**) Representative protein bands of immunoblot analysis for AMPA (GluA1 and GluA2) and NMDA (GluN1, GRIN2A, and GRIN2B). (**b2**) Quantification of AMPA (GluA1 and GluA2) and NMDA (GluN1, GRIN2A, and GRIN2B) receptor expression. (**c1**) Representative protein bands of immunoblot analysis for PSD-95. (**c2**) Quantification of PSD-95 expression. The data are expressed as the means ± SE (*n* = 4 to 8 mice/group). */^#^ *P* < 0.05; **/^##^ *P* < 0.01; ***/^###^ *P* < 0.001. Con = control group. Veh = vehicle control; Low = 1 mg/kg PM_2.5_; High = 5 mg/kg PM_2.5_; High PM_2.5_ = 5 mg/kg PM_2.5_

Synaptic failure is the cellular basis for cognitive impairment and is largely reflected by impaired long-term synaptic plasticity in terms of long-term potentiation (LTP). To clarify whether PM_2.5_ exposure affects long-term synaptic plasticity following BACE1 elevation, we measured LTP at the perforant path synapses in the dentate gyrus (DG) of the hippocampus. As shown in Fig. 3a, LTP was impaired in mice following 5 mg/kg bw PM_2.5_ exposure. Considering that the glutamate receptor is responsible for excitatory synaptic integrity, we further detected the expression of NMDA and AMPA receptor subunits (GluA1, GluA2, GluN1, GRIN2A, and GRIN2B). As shown in Fig. 3b, the expression of glutamate receptor subunits was reduced with increasing aspiration dose, with statistically significant differences between controls and exposure to PM_2.5_ at 1 and 5 mg/kg bw for GluA2 and at 5 mg/kg bw for GluA1, GluN1, GRIN2A, and GRIN2B. Additionally, we found that expression of postsynaptic density protein 95 (PSD-95), a postsynaptic marker, was statistically significantly reduced (Fig. 3c), and the synaptic ultrastructures of hippocampi also showed a thinned PSD and reduced synaptic vesicle density (Additional file 1: Figure S1). These results indicate increased BACE1 expression, as well as deterioration in functional synaptic integrity and spatial learning and memory in PM_2.5_-exposed animals.

### BACE1 inhibition maintains functional synaptic integrity and improves spatial learning and memory after PM_2.5_ aspiration

**Fig. 4:**
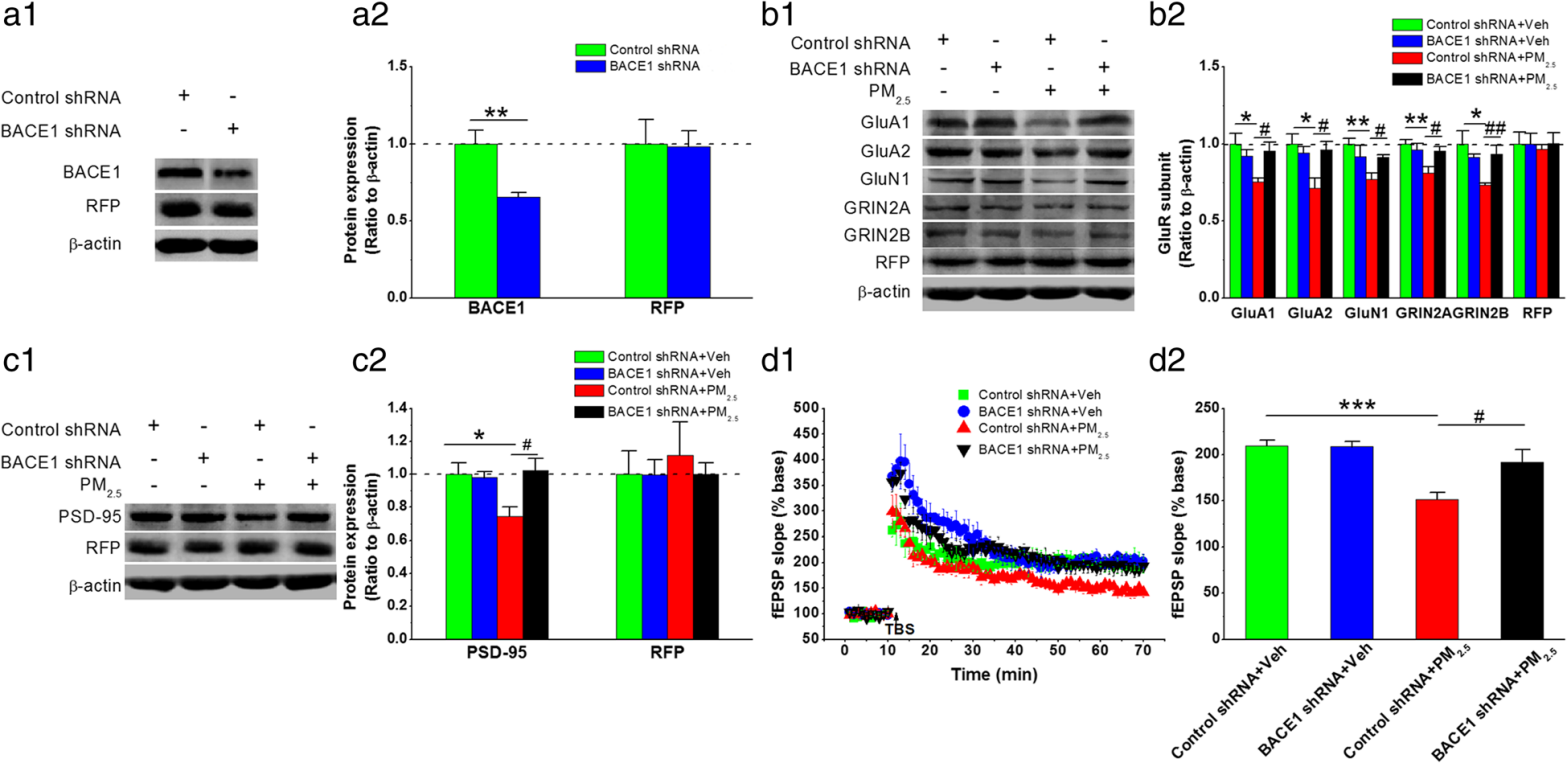
BACE1 inhibition maintains functional synaptic integrity following PM_2.5_ aspiration. (**a1**) Representative protein bands of immunoblot analysis for BACE1 and red fluorescent protein (RFP). (**a2**) Quantification of BACE1 and RFP expression. (**b1**) Representative protein bands of immunoblot analysis for AMPA (GluA1 and GLuA2), NMDA (GluN1, GRIN2A, and GRIN2B) and RFP. (**b2**) Quantification of AMPA (GluA1 and GluA2), NMDA (GluN1, GRIN2A, and GRIN2B) and RFP expression. (**c1**) Representative protein bands of immunoblot analysis for PSD-95 and RFP expression. (**c2**) Quantifications of PSD-95 and RFP expression. RFP expression was used as a marker to indicate viral infection in the immunoblot assay. The data are expressed as the means ± SE (*n* = 6 to 8 mice/group). (**d1**) Time courses of changes in the field excitatory postsynaptic potential (fEPSP) slope under different treatments. (**d2**) Mean values of the fEPSP slope averaged from 56 to 60 min after theta burst stimulation (TBS). The data are expressed as the means ± SE (*n* = 6 mice/group). */^#^ *P* < 0.05; **/^##^ *P* < 0.01; ***/^###^ *P* < 0.001

If sustained BACE1 elevation following PM_2.5_ exposure contributes to impairments in functional synaptic integrity and spatial learning and memory, then BACE1 inhibition should eliminate or attenuate these impairments. To test this hypothesis, we silenced BACE1 expression by stereotaxically injecting a lentivirus vector (LV) expressing BACE1 shRNA into the hippocampal area (Fig. 4a), and we subsequently determined whether BACE1 inhibition diminished the reduced expression of glutamate AMPA and NMDA receptor subunits after PM_2.5_ aspiration. The results indicated that GluA1, GluA2, GluN1, GRIN2A, and GRIN2B expression was significantly reduced in response to PM_2.5_ exposure at 5 mg/kg bw, and the reduction was attenuated or prevented by genetic BACE1 inhibition (Fig. 4b). Meanwhile, PM_2.5_-attenuated expression of PSD-95 (Fig. 4c) and abnormal synaptic ultrastructures (Additional file 1: Figure S2) were rescued by BACE1 silencing. Consistently, BACE1 inhibition also prevented LTP deterioration induced by PM_2.5_ at the perforant path synapses (Fig. 4d).

**Fig. 5:**
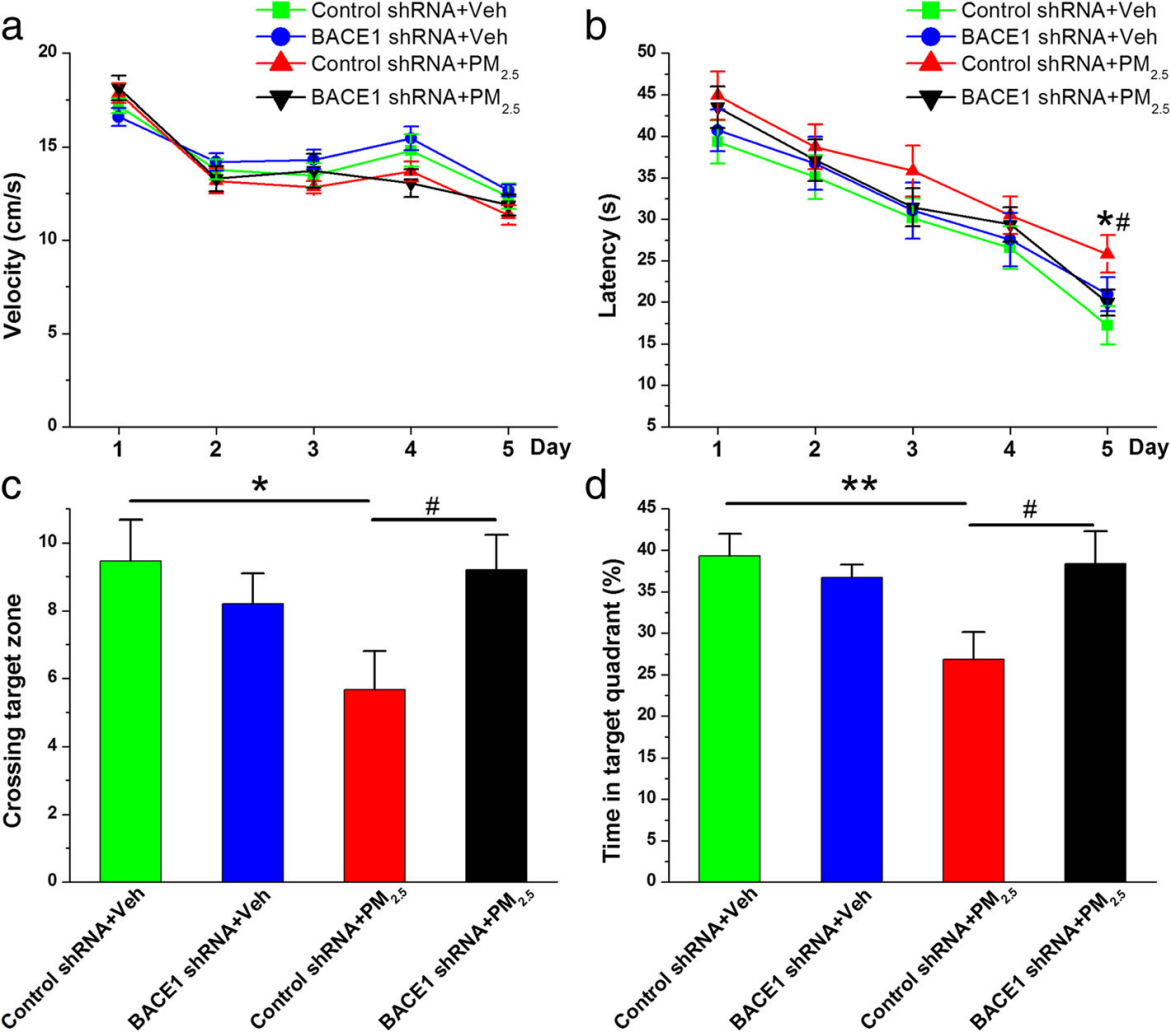
BACE1 inhibition improves spatial learning and memory following PM_2.5_ aspiration. **a** Swim velocity over 5 days of invisible training to find the hidden platform in the Morris water maze. **b** Learning curve over 5 days of invisible training to find the hidden platform in the Morris water maze. **c** Number of times crossing the target zone. **d** Percentage of time spent in the target quadrant. The data are expressed as the means ± SE (*n* = 13 to 14 mice/group). */^#^ *P* < 0.05; **/^##^ *P* < 0.01

PM_2.5_ exposure deteriorates learning and memory functions. If this action is associated with BACE1 elevation, then BACE1 inhibition should restore the impairment. To test the implications of these findings, we determined the effect of genetic BACE1 inhibition on spatial learning and memory in animals treated with PM_2.5_. As shown in Fig. 5a-d, genetic BACE1 inhibition diminished the deterioration in spatial learning and memory associated with PM_2.5_ aspiration. These findings suggest that BACE1 plays a critical role in synaptic and cognitive deterioration following PM_2.5_ exposure.

### PM_2.5_ exposure downregulates the expression of miR-574-5p which has a binding site in the 3′UTR of BACE1

**Fig. 6:**
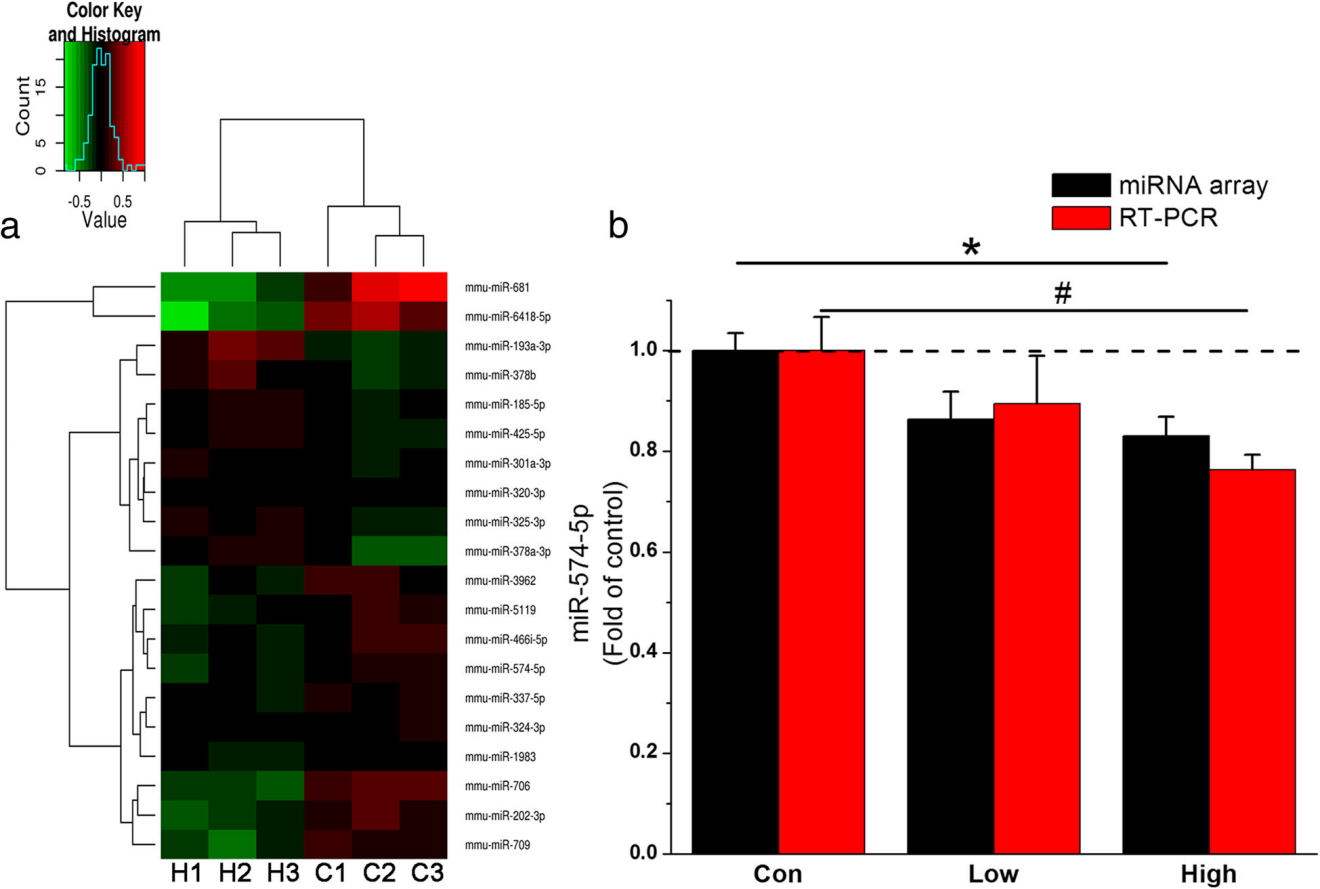
PM_2.5_ exposure downregulates miR-574-5p expression. (**a**) Hierarchical clustering of miRNAs detected in the hippocampus of C57BL/6 J mice exposed to control or 5 mg/kg bw PM_2.5_ (*n* = 3 mice/group). Colors represent expression levels of each individual miRNA: *black*, mean vector for all miRNA expression levels; *red*, upregulation compared with mean vector; green, downregulation compared with mean vector. “C1”, “C2” and “C3” repsents three individual mice from control group, and “H1”, “H2” and “H3” repsents three individual mice from high dose group, respectively. (**b**) Significant changes in miR-574-5p levels detected via microarray and qPCR. The data are presented as the means ± SE (*n* = 6 to 8 mice/group). */^#^ *P* < 0.05

To determine the molecular mechanism by which PM_2.5_ augmented BACE1 expression and produced deficits in synaptic transmission and spatial learning and memory, microarray was used as initial screening using unadjusted *p* < 0.05, with expression of relevant candidates verified by real-time qPCR. Compared with their expression in the control, 20 miRNAs were upregulated or downregulated in response to PM_2.5_ treatment at 5 mg/kg bw (Fig. 6a, *P* < 0.05). Among these altered miRNAs, miR-574-5p was homologous to the corresponding human miRNA, and the significantly downregulated expression was validated by miRNA real-time qPCR (Fig. 6b). Considering its relationship with central nervous system development and that mediation of post-transcriptional regulation [50] and dysregulation of miR-574-5p have been reported in neurodegenerative diseases [51, 52], we focused on miR-574-5p and examined its predicted binding sites in the 3′ untranslated region (UTR) of BACE1. Our results showed that miR-574-5p expression was decreased to 0.89- and 0.76-fold of the control after PM_2.5_ exposure at 1 and 5 mg/kg bw, respectively, with a statistical significant difference being observed at the higher dose.

**Fig. 7:**
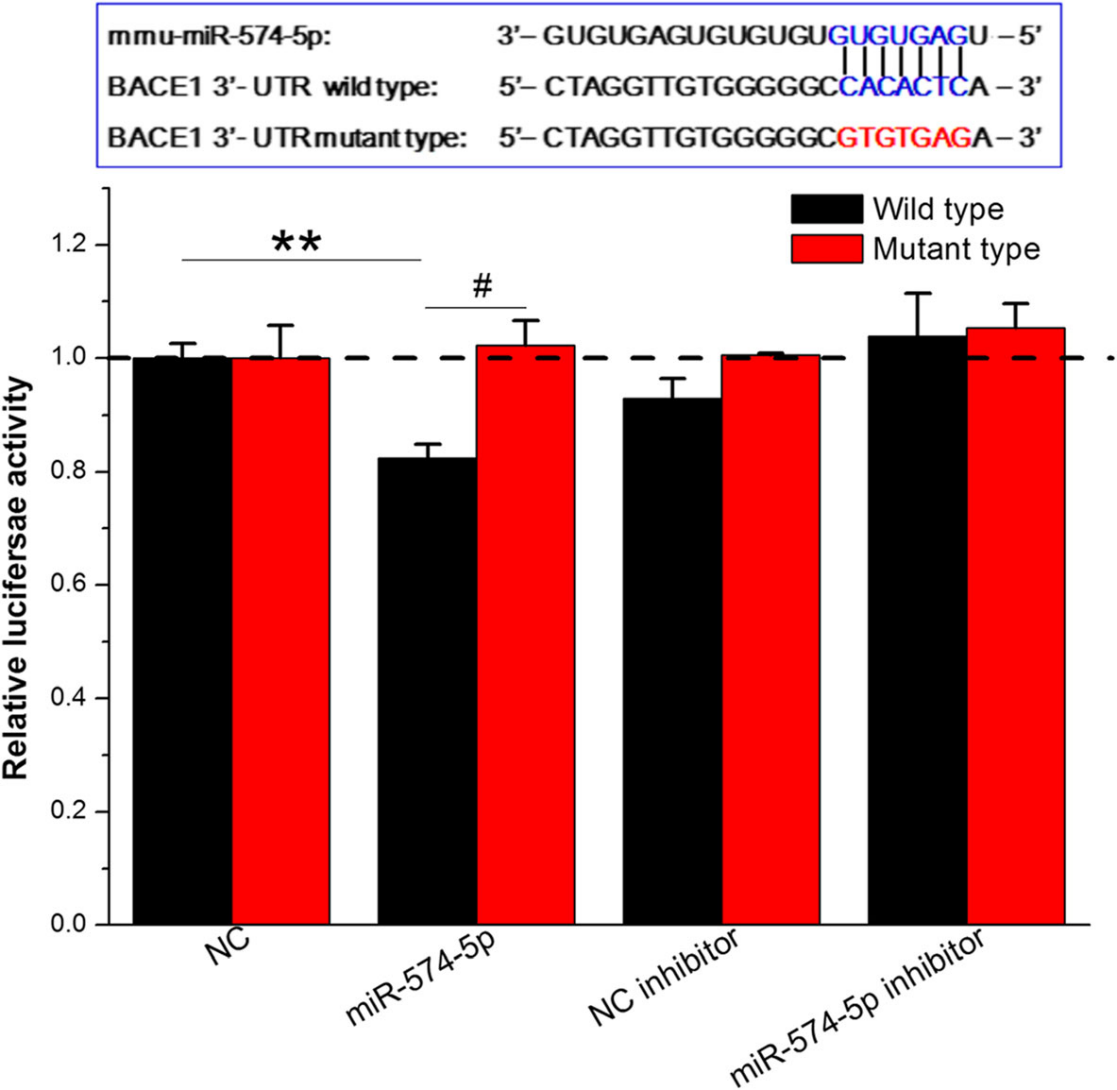
Direct inhibition of BACE1 expression by miR-574-5p was detected with a dual-luciferase-luciferase reporter system. BACE1 contains a conserved 3′-UTR sequence (positions 1458–1464) that perfectly complements the miR-574-5p seed sequence (both are shown in *blue*). The mutant BACE1 3′-UTR contains mutations in the miR-574-5p binding site that disrupt base pairing (indicated in *red*). The data regarding the different effects of miR-574-5p on the BACE1 3′-UTR and its mutant are presented as the means ± SE (*n* = 6). */^#^ *P* < 0.05; **/^##^ *P* < 0.01. NC = negative control

According to the statistical analysis, miR-574-5p has at least one binding site in the 3’UTR of BACE1. To functionally verify the predicted binding site, we inserted the entire sequence of the mouse 3’UTR of BACE1 into a dual-luciferase reporter constructs. Figure 7 shows that miR-574-5p efficiently inhibited luciferase expression by binding to the BACE1 3′-UTR and significantly reduced the relative luciferase reporter activity of the wild-type BACE1 3′-UTR, whereas the luciferase reporter activity of the mutant BACE1 3′-UTR was not altered, suggesting that miR-574-5p could directly bind to the 3′-UTR of BACE1 but did not inhibit the reporter activity with the mutated luciferase construct. This finding suggests that reduced expression of miR-574-5p, via altered binding to the 3’UTR of BACE1, may be conducive to BACE1 elevation and synaptic and cognitive impairment following PM_2.5_ exposure.

### miR-574-5p overexpression suppresses BACE1 elevation and restores synaptic and cognitive impairment following PM_2.5_ exposure

**Fig. 8:**
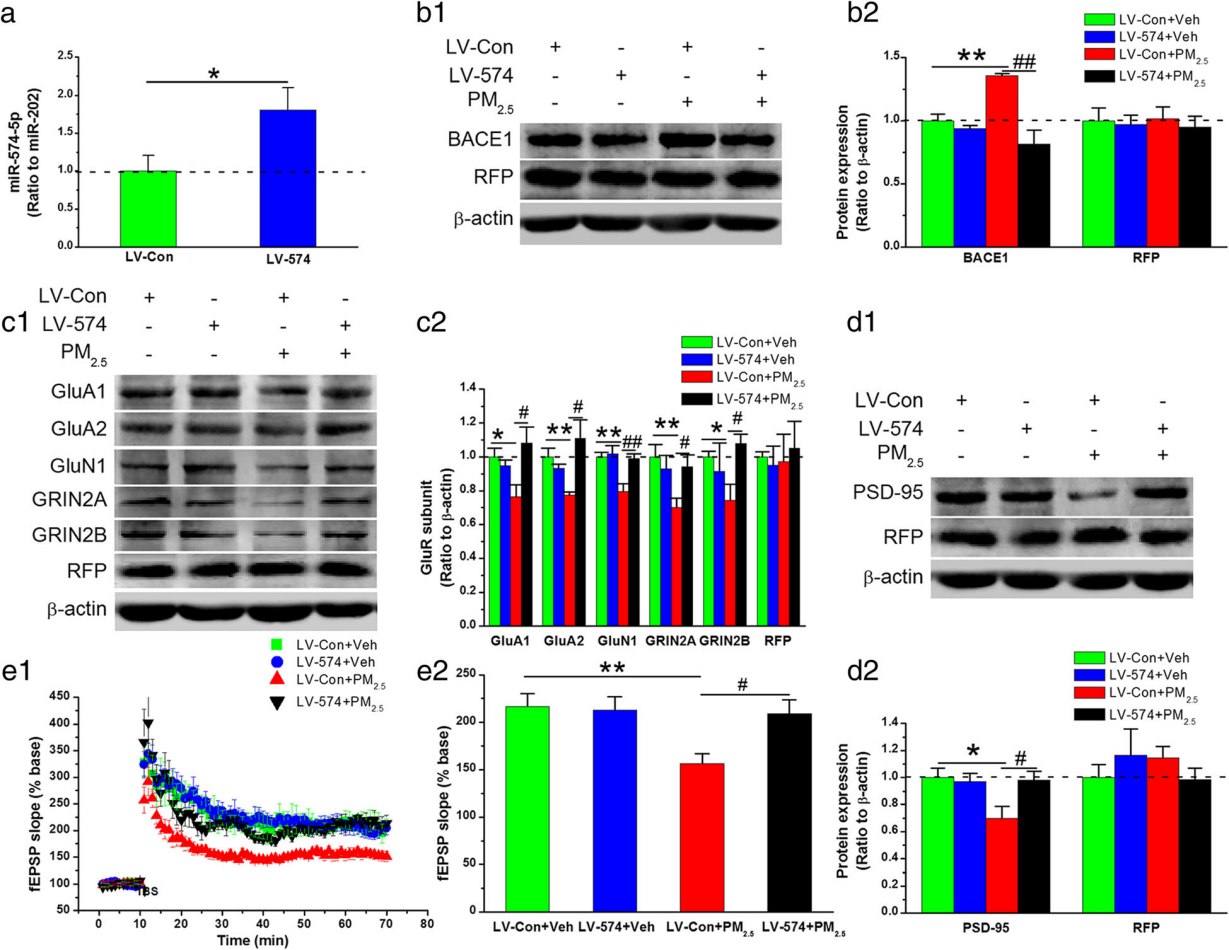
miR-574-5p overexpression suppresses BACE1 expression and restores synaptic impairment following PM_2.5_ exposure. (**a**) Significant changes in miR-574-5p expression were detected via qPCR. (**b1**) Representative protein bands of immunoblot analysis for BACE1 and RFP. (**b2**) Quantification of BACE1 and RFP expression. (**c1**) Representative protein bands of immunoblot analysis for AMPA (GluA1 and GLuA2), NMDA (GluN1, GRIN2A, and GRIN2B) and RFP. (**c2**) Quantification of AMPA (GluA1 and GLuA2), NMDA (GluN1, GRIN2A, and GRIN2B) and RFP expression. (**d1**) Representative protein bands of immunoblot analysis for PSD-95 and RFP. (**d2**) Quantification of PSD-95 and RFP expression. The data are expressed as the means ± SE (*n* = 6 to 8 mice/group). (**e1**) Time courses of changes in the field excitatory postsynaptic potential (fEPSP) slope under different treatments. (**e2**) Mean values of the fEPSP slope averaged from 56 to 60 min after theta burst stimulation (TBS). The data are expressed as the means ± SE (*n* = 6 mice/group). */^#^ *P* < 0.05; **/^##^ *P* < 0.01. LV-Con + Veh = LV-scramble control + vehicle; LV-574 + Veh = LV-miR-574-5p + vehicle; LV-Con + PM_2.5_ = LV-scramble control + PM_2.5_; LV-574 + PM_2.5_ = LV-miR-574-5p + PM_2.5_

If miR-574-5p directly binds to the 3’UTR of BACE1 and miR-574-5p deregulation stimulates BACE1 expression and impairs synaptic and cognitive function in response to PM_2.5_ aspiration, then overexpression of miR-574-5p should suppress BACE1 elevation and restore synaptic and cognitive impairment. To test this prediction, we overexpressed miR-574-5p by stereotaxically injecting LV into the hippocampal area (Fig. 8a), and detected BACE1 expression in the absence or presence of PM_2.5_ aspiration. With PM_2.5_ treatment, miR-574-5p overexpression statistically significantly attenuated BACE1 elevation, in contrast to that observed in LV-scramble control mice (Fig. 8b). These results indicate that miR-574-5p overexpression successfully suppressed BACE1 expression after PM_2.5_ aspiration.

**Fig. 9:**
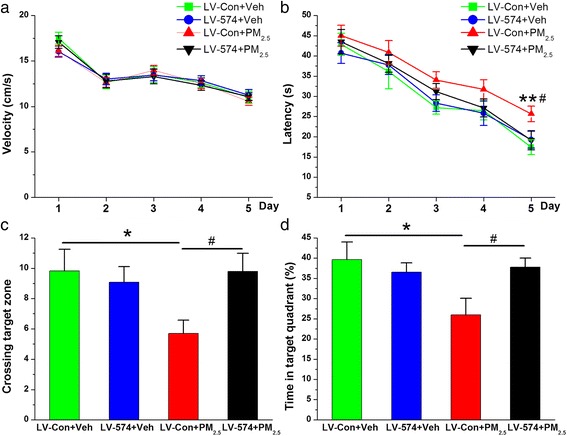
miR-574-5p overexpression relieves cognitive deterioration in response to PM_2.5_ aspiration. **a** Swim velocity over 5 days of invisible training to find the hidden platform in the Morris water maze. **b** Learning curve over 5 days of training to find the hidden platform. **c** Number of times crossing the target zone. **d** Percentage of time spent in the target quadrant. The data are expressed as the means ± SE (*n* = 12 to 14 mice/group). */^#^ *P* < 0.05; **/^##^ *P* < 0.01. LV-Con + Veh = LV-scramble control + vehicle; LV-574 + Veh = LV-miR-574-5p + vehicle; LV-Con + PM_2.5_ = LV-scramble control + PM_2.5_; LV-574 + PM_2.5_ = LV-miR-574-5p + PM_2.5_

Following the above results, we further determined whether overexpression of miR-574-5p could reverse synaptic and cognitive deterioration. Here, we measured glutamate NMDA and AMPA receptor expression, LTP, and spatial learning and memory in mice that received either LV-miR-574-5p or LV-scramble in the absence or presence of PM_2.5_ treatment. In response to PM_2.5_ aspiration, the reduced expression of the glutamate NMDA and AMPA receptor subunits and PSD-95 was statistically significantly restored in animals treated with LV-miR-574-5p compared with that in those treated with the LV-scramble control (Fig. 8c and d). Furthermore, we found that the abnormal synaptic ultrastructures (Additional file 1: Figure S3) and reduced LTP (Fig. 8e) recovered following miR-574-5p overexpression. Importantly, PM_2.5_-exposed mice showed improved behavioral performance after receiving LV-miR-574-5p in contrast to those receiving the LV-scramble control, including an effectively reversed prolonged latency of reaching the platform, a significantly increased number of times crossing the target zone, and an increased amount of time spent in the target quadrant (Fig. 9). These data provide further evidence that miR-574-5p mediates PM_2.5_-induced BACE1 elevation following synaptic and cognitive deterioration.

### NF-κB activation regulates miR-574-5p expression in response to PM_2.5_ exposure

**Fig. 10:**
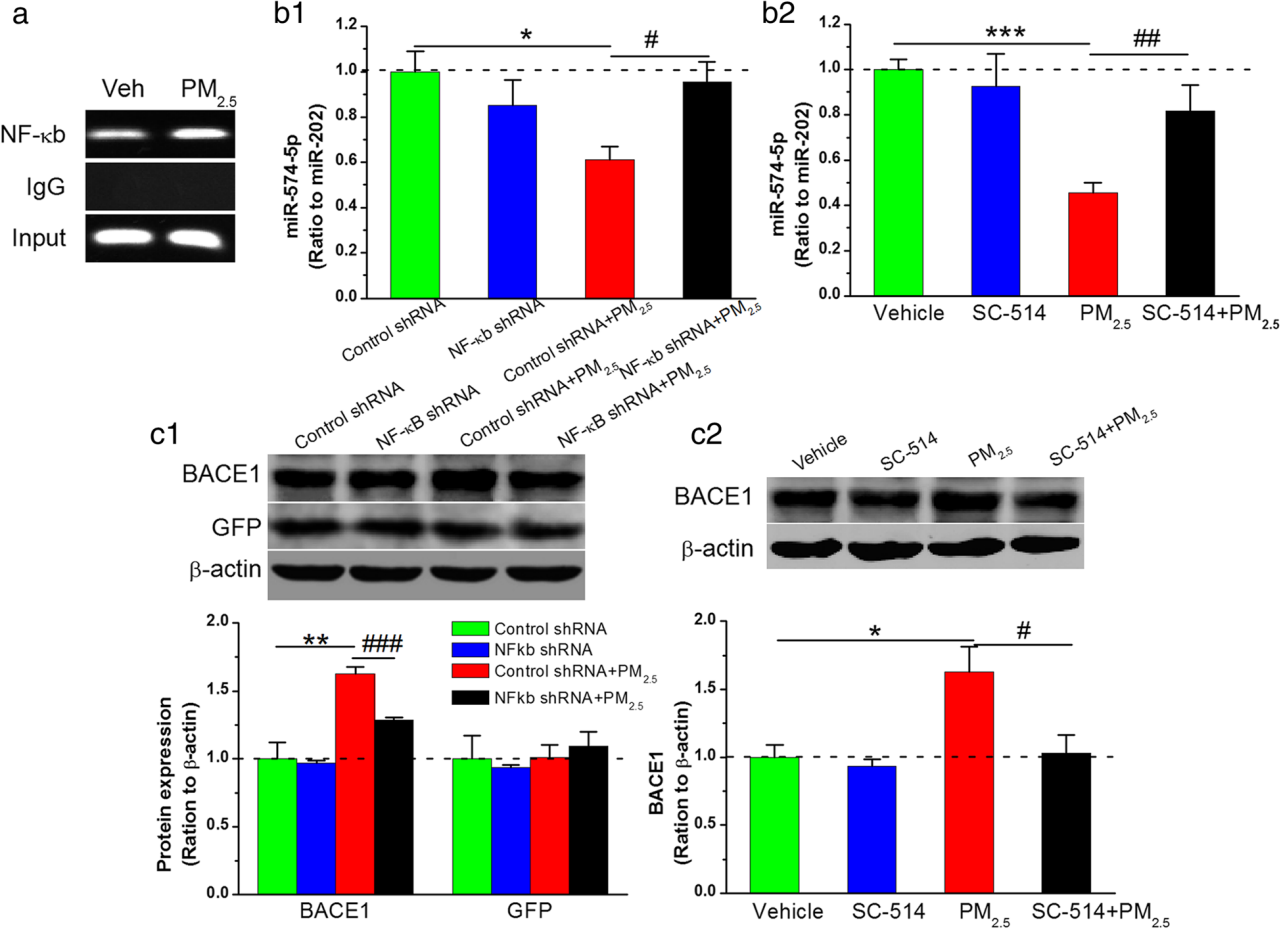
NF-κB activation regulates miR-574-5p expression in response to PM_2.5_ exposure. (**a**) Binding of the NF-κB p65 subunit to the promoter of the miR-574-5p gene. IgG was used as a negative control in the chromatin immunoprecipitation assay. (**b1**) Knockdown of NF-κB p65 using a shRNA silencing technique eliminates the reduction in miR-574-5p following 10 μg/mL PM_2.5_ treatment in primary cultured hippocampal neurons. (**b2**) Knockdown of NF-κB p65 by applying SC-514 (100 μM), an IKKβ specific inhibitor, eliminates the reduction in miR-574-5p following 10 μg/mL PM_2.5_ treatment in primary cultured hippocampal neurons. (**c1**) Knockdown of NF-κB p65 using a shRNA reverses the elevation in BACE1 in response to 10 μg/mL PM_2.5_ treatment in primary cultured hippocampal neurons. (**c2**) Knockdown of NF-κB p65 by applying SC-514 restores the BACE1 elevation in response to 10 μg/mL PM_2.5_ treatment in primary cultured hippocampal neurons. Green fluorescent protein (GFP) expression was used as a marker to indicate viral infection in the immunoblot assay. The data are expressed as the means ± SE (*n* = 4 to 8). */^#^ *P* < 0.05; **/^##^ *P* < 0.01; ***/^###^ *P* < 0.001

miR-574-5p is a previously unrecognized miRNA that targets BACE1 following PM_2.5_ aspiration. Therefore, it was necessary to clarify how miR-574-5p is regulated following exposure. NF-κB is an important transcription factor that regulates the expression of a variety of miRNAs [40, 53] and is involved in neurotoxicity upon chronic exposure to ozone, PM or a myriad of other air pollutants [54, 55]. Indeed, others have reported that mice exposed to concentrated PM_2.5_ for 2 weeks show neuroinflammatory cytokine-coupled NF-κB activation [56]. Thus, it was essential to clarify whether and how NF-κB is linked to miR-574-5p deregulation in response to PM_2.5_ aspiration. To address this issue, we first used PROMO (http://alggen.lsi.upc.es/cgi-bin/promo_v3/promo/promoinit.cgi?dirDB=TF_8.3) to predict potential binding sites between NF-κB p65 and miR-574-5p, and the results showed that there was at least one NF-κB binding site. Importantly, by designing PCR amplicons, the binding site was validated via chromatin immunoprecipitation (ChIP) analysis. As presented in Fig. 10a, binding activity of NF-κB p65 was detected in the promoter positions of miR-574-5p, and this interaction was enhanced by PM_2.5_ treatment. Additionally, through the dual-luciferase reporter system in HEK293T cells, we found that PM_2.5_ directly stimulated NF-κB activity (Additional file 1: Figure S4), suggesting that downregulation of miR-574-5p by PM_2.5_ likely occurs through activating NF-κB and increasing the reverse modulation of NF-κB on miR-574-5p. If NF-κB activation regulates miR-574-5p expression and then induces BACE1 elevation in response to PM_2.5_ exposure, then NF-κB inhibition should rescue the reduced miR-574-5p expression and elevated BACE1 activation. To verify this notion, we pharmacologically and genetically inhibited NF-κB and detected miR-574-5p expression and BACE1 activation by applying an NF-κB inhibitor, SC-514, and using an NF-κB p65 shRNA silencing technique in primary cultured hippocampal neurons. Our findings revealed that inhibition of NF-κB diminished PM_2.5_-induced repression of miR-574-5p and induction of BACE1, which further supports our earlier results (Fig. 10b and c).

## Discussion

The results of our present study provide evidence that PM_2.5_ aspiration impairs synaptic and cognitive function and that this effect is associated with a previously unrecognized role for BACE1 induction through NF-κB signaling-mediated miR-574-5p downregulation. Importantly, BACE1 inhibition rescued synaptic and cognitive impairment in animals following PM_2.5_ aspiration, which was further supported by the fact that overexpression of miR-574-5p robustly reduced BACE1 elevation and rescued synaptic and cognitive impairment. Additionally, PM_2.5_-induced downregulation of miR-574-5p expression and upregulation of BACE1 were reversed by NF-κB inhibition.

The concern over PM targeting the brain was first reported by Oberdörster et al. [57] and further strengthened by Maher et al. [58]. Generally, this process is thought to occur through two pathways. On the one hand, sustained exposure to fine particles and ultrafine particles may result in direct translocation of particles to systemic circulation and the brain, and the nasal olfactory pathway is believed to be a key portal of entry. On the other hand, PM could trigger the release of inflammatory mediators from primary entry organs or secondary deposition sites, and these inflammatory agents may lead to or alter the susceptibility to neuroinflammation and neurodegeneration in the brain. Here, we found that PM_2.5_ aspiration dose-dependently increased IL-1β and TNF-α levels in the lungs, blood and brain. Importantly, no significant changes were observed following exposure to an inert particle, black carbon, at the same exposure levels. Although this does not exclude the possibility of direct particle translocation, it is possible that PM_2.5_ triggers the release of soluble inflammatory mediators from the lungs and subsequently causes neuroinflammation through systemic inflammatory responses. Consistent with our results, recent studies from Mexico City have reported that air pollutants including PM caused neuroinflammatory responses and promoted AD-like pathologies [59, 60]. Additionally, our previous studies and those of others showed that PM_2.5_, individually or in combination with other components, can affect brain structure, cause synaptic neurotoxicity, promote neurodegeneration and deteriorate synaptic and cognitive function [61–64]. However, the molecular mechanisms underlying these adverse effects are largely unknown.

BACE1 is not only a dominant enzyme in Aβ synthesis from APP but also functions as an aspartic protease with altered enzymatic activity and protein expression in the brain early in the development of mild cognitive impairment [48]. Other studies have reported that PM_2.5_-increased Aβ production was coupled with BACE1 elevation [27]. In the present study, our results showed that PM_2.5_ aspiration increased APP and BACE1 expression but not Aβ accumulation or deposition. The transmembrane protein APP can be processed by two pathways: the nonamyloidogenic α-secretase pathway and the amyloidogenic β-secretase pathway. In the nonamyloidogenic pathway, APP is cleaved by α-secretase and γ-secretase to release the soluble APP-fragment sAPP-α, the APP intracellular domain (AICD) and the P3 fragment. In the amyloidogenic pathway, APP is cleaved by β-secretase and γ-secretase to produce the soluble fragment sAPP-β, Aβ40, Aβ42 and AICD. To clarify the above results, we further detected the expression of α-secretase (ADAM10) and γ-secretase (nicastrin) and did not observe significant alterations. Aβ deposition is slow and protracted, and the slow rate of Aβ deposition indicates a wide time window for altering Aβ accumulation [65]. Klunk et al. demonstrated a schematic of three hypothetical phases of Aβ amyloid deposition as follows: (i) very early initiation; (ii) continuously progressive; and (iii) late equilibrium/symptomatic [66]. In the present study, nicastrin expression and Aβ42 deposition were not increased following PM_2.5_ exposure for 4 weeks; however, we cannot exclude the possibility that the time point was too early to see significant alterations. Previous studies have shown that BACE1-catalyzed APP cleavage exerted positive effects on cognitive function, and AICD, which is correlated with the promotion of synaptic transmission and memory performance, is a potential mediator during this process [67]. Our present results revealed that PM_2.5_ exposure resulted in decreased expression of glutamate AMPA and NMDA receptor subunits and LTP and spatial learning and memory impairment in normal wild-type animals. Similar to our results, other studies have documented that PM_2.5_ altered synaptic gene expression and caused synaptic and cognitive dysfunction [16, 27, 28]. Indeed, increased BACE1 activity may affect normal synaptic functioning given that, in addition to Aβ, there are other BACE1 cleavage products [49]. Changes in the normal function of the synapse may result in the neurochemical deficits and behavioral abnormalities that have been reported in BACE1 transgenic models [68, 69]. Importantly, here, we show that genetic inhibition of BACE1 reversed PM_2.5_-induced attenuation of AMPA and NMDA receptor subunit expression and impairment of LTP and spatial learning and memory. Although we did not clarify the polypeptide produced by BACE1-mediated cleavage of APP, these findings confirm that BACE1 plays an important role in PM_2.5_-mediated synaptic and cognitive impairment and suggest the possibility that further study of BACE1 regulation may provide insight into the underlying signaling pathways mediating harmful outcomes.

Considering the vital role of miRNAs in post-transcriptional regulation, their widespread expression in different brain regions and their regulation by multiple mechanisms, miRNAs are increasingly considered to be central players in synaptic plasticity, a critical mechanism that is thought to underlie many complex brain functions, including learning and memory [70]. Indeed, it has been reported that multiple miRNAs participate in synaptic and cognitive impairment and AD-like neuropathology, including miR-9, miR-34, miR-132, miR-137, miR-188, miR-204, miR-211, and miR-212 [71–77]. In the brains of sporadic AD patients, an increase in BACE1 levels is correlated with a decrease in a subset of miRNAs [78]. Therefore, it is possible that a decrease in specific miRNA levels may upregulate BACE1 expression, resulting in synaptic and cognitive impairment. Indeed, several miRNAs target BACE1, including miR-9, miR-29, miR-107, miR-186, miR-188, miR-298, and miR-328, some of which are closely related to synaptic and cognitive function [40, 41]. A recent study reported that long-term exposure to black carbon was linked to cognitive status due to influences on miRNA expression [79]. However, little is known regarding whether miRNAs aggravate synaptic and cognitive dysfunction by elevating the expression of BACE1 following PM_2.5_ exposure. To address this question, we performed a miRNA microarray analysis and found that miR-574-5p, which is homologous in humans and targets BACE1, was downregulated in a dose-dependent manner following PM_2.5_ exposure, suggesting that BACE1 elevation following PM_2.5_ exposure is likely the result of reduced miR-574-5p expression. If this hypothesis is correct, restoring or reversing these downregulated miRNAs in animal models following PM_2.5_ exposure should ameliorate synaptic and cognitive impairment by reducing BACE1 expression. Indeed, our results showed that overexpression of miR-574-5p significantly reduced BACE1 elevation, which supported this speculation. Importantly, miR-574-5p overexpression significantly restored the attenuation of glutamate receptor expression and the deterioration of LTP and spatial learning and memory in response to PM_2.5_ aspiration. This information provides further evidence that the adverse effects of BACE1-mediated synaptic and cognitive deterioration resulting from PM_2.5_ aspiration are associated with downregulation of miR-574-5p through its binding to the 3’UTR of BACE1.

Numerous investigations have reported a role of miRNAs in physiological functions, such as immune responses, cell proliferation, cell death, and inflammation, which are also known to be regulated by NF-κB [80]. Recent research has also revealed downregulation of miR-188-3p in AD patients and APP transgenic animals, which was directly regulated by NF-κB [40]. More importantly, PM causes neuroinflammatory responses by stimulating pro-inflammatory cytokine release, which is associated with NF-κB activation [81, 82]. Here, to provide a more complete picture of the different aspects of miRNA modulation after PM_2.5_ exposure, we used ChIP analyses to show that NF-κB p65 was able to bind to the promoter region of the miR-574-5p gene. Additionally, PM_2.5_ increased NF-κB reporter activity, indicating that NF-κB activation following PM_2.5_ exposure amplifies NF-κB-mediated inhibition of miR-574-5p transcription. Importantly, pharmacological and genetic silencing of NF-κB p65 prevented the miR-574-5p reduction and BACE1 elevation following PM_2.5_ exposure, supporting our hypothesis. The PM_2.5_-induced increase in BACE1 expression not fully rescued by NF-κB shRNA may be due to several transcription factors have interactions with BACE1 except NF-κB. For instance, specificity protein 1 (Sp1) plays an important role in regulation of BACE1 to process APP generating Aβ in AD [83]. In addition, there may be the compensatory mechanism after knockdown of NF-κB. Together, our results suggest that PM_2.5_ aspiration suppresses miR-574-5p expression by augmenting NF-κB activity, which then facilitates BACE1 activation and results in synaptic and cognitive impairment.

## Conclusion

The present study confirms that PM_2.5_ exposure leads to impaired synaptic and cognitive function, and this action is associated with a previously unrecognized role for BACE1 induction through NF-κB signaling-mediated miR-574-5p downregulation. The results suggest a novel mechanism for PM_2.5_-produced adverse effects on the nervous system and present a potential intervention target for prevention.

## Methods

### Particle preparation and characterization

The PM_2.5_ sampling point was located in Taiyuan (112°21–34′E longitude, 37°47–48′N latitude) of Shanxi Province. The samples were collected onto quartz filters (Φ90 mm, Munktell, Falun, Dalarna, Sweden) with PM middle-volume air samplers (TH-150CIII, Wuhan, China) from November 2012 to February 2013 according to our previously reported conditions [84], and the PM characterization was presented in our recently published work (Additional file 1: Table S1) [85]. The collected PM_2.5_ was transferred into aqueous suspension by soaking the PM_2.5_-loaded filters in Milli-Q deionized water for 30 min, followed by vortexing (5 min) and sonication (30 min). Prior to use, the dried sample was diluted with sterilized saline and then swirled and sonicated for 10 min.

Printex u was purchased from Degussa Ltd. (Frankfurt, Germany) and utilized as black carbon powder. The size of its particle was 30 nm under the electron microscope and the specific surface area was 97.63 m^2^/g. The particles were suspended in sterilized saline and then swirled and sonicated for 10 min before oropharyngeal aspiration.

### Animal treatment

Male C57BL/6 mice, approximately 8 weeks old, were obtained from the Experimental Animal Center at the Academy of Military Medical Sciences of Chinese PLA (Beijing, China) and housed under standard conditions. Considering the actual population exposure dose, we chose the administration dose of 1 and 5 mg/kg bw PM_2.5_ in mice. According to Grade II PM_2.5_ in China, the amount of PM_2.5_ exposure at 0.075 mg/m^3^ for 2 days is 0.019 mg. As previously mentioned, the respiratory volume of the mice was 90 mL/min (Gurkan et al., 2003) and the respiratory volume was 0.259 m^3^ for 2 days. Thus, the PM_2.5_ exposure dose in mice should be 0.95 mg/kg bw every 2 days. However, the average concentration of PM_2.5_ in northern China with non-haze weather was 0.161 mg/m^3^ [86], and the level with haze weather reached 0.692 mg/m^3^ [87]. In our study, the lower PM_2.5_ dose used was 1 mg/kg bw, within the range of the second standard in Chinese ambient air quality regulation, and the higher PM_2.5_ exposure dose used for mice was 5 mg/kg bw, which was 5-fold higher than that in Grade II PM_2.5_ in China but still in the range of the reported maximum PM_2.5_ levels.

In the PM_2.5_ treatment groups, animals received oropharyngeal aspiration of PM_2.5_ (1 and 5 mg/kg bw) every other day for 4 weeks, with each group administered 20 or 100 μg of PM_2.5_ in 20 μL of saline. In the vehicle control group, mice were treated with the same amount of saline prepared by ultrasonic oscillation of the control membrane filter using the same protocol. In the control group, mice received oropharyngeal aspiration of saline every other day for 4 weeks. In the black carbon treatment groups, animals received oropharyngeal aspiration of black carbon (5 mg/kg bw) every other day for 4 weeks, with each group administered 100 μg of black carbon in 20 μL of saline. To provide direct evidence that BACE1 and miR-574-5p were involved in the neurological effects observed after PM_2.5_ treatment, animals were stereotaxically injected with lentiviral constructs encoding BACE1 shRNA and control shRNA or LV-miR-574-5p and LV-scramble control for interference measurement. In detail, mice received a bilateral microinjection (5 μL at 0.2 μL/min/side) into the hippocampus (anteroposterior, 2 mm; mediolateral, ± 1.8 mm; and dorsoventral, −2 mm). After 6 weeks, four groups (control shRNA, BACE1 shRNA, LV-scramble control and LV-miR-574-5p) received an oropharyngeal aspiration of 5 mg/kg PM_2.5_ or saline, which was processed by ultrasonic oscillation of the control membrane filter, every other day for 4 weeks. Before PM_2.5_ or saline administration, the mice were anesthetized with isoflurane (Yi Pin Pharmaceutical Co., Ltd., Hebei, China). When not being treated, the animals had free access to water and standard feed. Mice were sacrificed 18 h after the final exposure. The hippocampal tissues, lungs and blood (with no interference treatment) were collected, quickly frozen in liquid nitrogen, and stored at −80 °C. The mice were treated humanely according to the National Institutes of Health Guide for the Care and Use of Laboratory Animals, and all animal experiments in this study were approved by the Institutional Animal Care and Use Committee of Shanxi University (Approved Animal Use Protocol Number: HZ20140503).

### Primary hippocampal neuron culture and treatment

Primary cultured hippocampal neurons were prepared according to a protocol previously reported [88]. Briefly, the hippocampus, dissected from a mouse pup at postnatal day 1, was mechanically triturated after being incubated in oxygenated trypsin, and the cells were spun down and resuspended in Neurobasal/B27 medium. Because of the complex activity of the NF-κB signaling pathway, it is impossible for us to interfere with its expression in vivo. Based on the literature, we conducted the experiment in vitro [40, 89], and cells (1 × 10^6^) were divided randomly into different groups. The control group was incubated only in Neurobasal/B27 medium, and the other groups were treated with 10 μg/ml PM_2.5_ for 24 h in the absence or presence of the NF-κB inhibitor SC-514 (100 μM) or were subjected to NF-κB silencing using small hairpin RNA (shRNA).

### MicroRNA microarray analysis

Agilent Mouse miRNA V19.0 was used for miRNA microarray analysis. The microarray data have been deposited in the NCBI Gene Expression Omnibus (GEO) database and are accessible through GEO Series accession number GSE93967. Briefly, total RNA was extracted and purified, and the integration was inspected according to previously described methods [90]. Then, miRNA was labeled and hybridized using a miRNA Complete Labeling and Hyb Kit (Agilent Technologies, Santa Clara, CA, USA) according to the manufacturer’s instructions. After hybridization, slides were washed in staining dishes (Thermo Shandon, Waltham, MA, USA) with a Gene Expression Wash Buffer Kit (Agilent Technologies) and scanned with an Agilent Microarray Scanner using Feature Extraction Software 10.7 (Agilent Technologies). Gene Spring Software 11.0 (Agilent Technologies) was used to analyze the raw data.

### Lentiviral constructs

The TargetScan (http://www.targetscan.org), PITA (http://genie.weizmann.ac.il/pubs/mir07/mir07_data.html), mirwalk (http://www.umm.uni-heidelberg.de/apps/zmf/mirwalk/) and miranda (http://www.microrna.org/microrna/home.do) websites were used to predict miRNA targets. The pLVX-IRES-TDtomato LV (Clontech) was used to insert mature miR-574-5p driven by the CMV promoter. pHelper 1.0 and pHelper 2.0 vectors were used for viral envelope production. pLVX-IRES-TDtomato LV was generated and packaged in HEK293T cells, and the transfection rate was measured by observing the expression of enhanced red fluorescent protein (RFP) under a fluorescence microscope. The average LV titer was 1.0 × 10^8^ infectious units/mL. pLVX-IRES-TDtomato LV was also used for BACE1 shRNA in C57BL/6 mice. The oligos for the BACE1 shRNA construct (5′-GACGCTCAACATCCTGGTG-3′) and scramble construct (5′-TTGGCTTTGCTGTCAGCGC-3′) were used for BACE1 knockdown experiments.

pLVX-shRNA2 LV (Clontech) was used for NF-κB p65 shRNA in primary cultured hippocampal neurons. The transfection rate was measured by observing the expression of enhanced green fluorescent protein (GFP) under a fluorescence microscope. Oligos for the NF-κB p65 shRNA construct (5′-AGGACCTATGAGACCTTCAAG-3′) and control shRNA (empty vectors) were used for knockdown of NF-κB p65. All of the constructs were verified by sequencing.

### Real-time quantitative reverse transcription PCR

Total RNA was extracted according to the manufacturer’s protocol using an miRNeasy Mini Kit (Qiagen Biotechnology Co., Ltd., Dalian) and then synthesized to complementary DNA (cDNA) using a reverse transcription kit (Qiagen Biotechnology, Germany). RT-PCR with specific primers was performed on a qTOWER 2.2 Real-Time PCR machine (Analytik Jena AG, Jena, Germany) according to the protocol for the miScript SYBR Green PCR Kit (Qiagen Biotechnology, Germany). Briefly, each 20-μL PCR reaction contained 2 μL of cDNA (2.5 ng/μL), 10 μL of 10× QuantiTect SYBR Green, 4 μL of RNase-free H_2_O, 2 μL of 10× miScript Universal Primer, and 2 μL of 5 μmol/L specific primers (Invitrogen). The reaction conditions for mmu-miR-574-5p and mmu-miR-202 were as follows: after 15 min at 95 °C, 40 cycles were performed at 95 °C for 20 s with respective annealing temperatures of 58.6 °C (mmu-miR-574-5p) and 55 °C (mmu-miR-202) for 20 s and then 72 °C for 30 s. The primer sequences were 5′-TGAGTGTGTGTGTGTGAGTGTGT-3′ for mmu-miR-574-5p and 5′-TTGAACCCTTTTCCATCTGA-3′ for mmu-miR-202.

### Dual-luciferase reporter gene analysis

To assess the interaction between miR-574-5p and BACE1 mRNA, the BACE1 3′-UTR was amplified by PCR using genomic DNA from mouse brain tissues and the following primers: 5′-CCGCTCGAGGGAGGCCCGTGGGCAGATGATGG-3′ (forward) and 5′-ATAAGAATGCGGCCGCCAGTGGAGATAGGTCAGTCATTTTTC-3′ (reverse). A construct possessing mutations disrupting the putative mir-574-5p binding site in the 3′-UTR of BACE1 was prepared using KOD Plus neo DNA Polymerase (ToYoBo) with the following primers: 5′-TGGTTCTTGGGCTAGGTTGTGGGGGGGTGTGAGACCTCTTCCCTGCCAGTTCTAACAC -3′ (forward) and 5′-GTGTTAGAACTGGCAGGGAAGAGGTCTCACACCCCCCCACAACCTAGCCCAAGAACCA-3′ (reverse). Both the wild-type and mutated plasmid clones were verified by DNA sequencing. The PCR products were cloned into the psiCHECK-2 vector (Promega) downstream of the luciferase gene. HEK293T cells were plated onto 24-well plates (2 × 10^4^/well) in DMEM containing 10% FBS 24 h before transfection. Using Lipofectamine 2000 (Invitrogen), cells were transfected with 100 ng/mL of either the wild-type or mutant 3′-UTR vector and 50 nM miR-574-5p mimic or 100 nM miR-574-5p inhibitor. At 48 h after transfection, luciferase activity was measured in cell lysates using a dual-luciferase reporter kit (Promega, Madison, WI, USA). HEK 293 T cells are a very useful tool for luciferase reporter assays and for lentiviral production and amplification [91, 92]. The effects of PM_2.5_ on NF-κB promoter activity were determined in HEK293T cells transfected with the pGL3-NF-κB promoter luciferase construct. At 24 h after transfection, cells were exposed to 10 μg/ml PM_2.5_ for 24 h, and then, luciferase activity was measured in cell lysates using a dual-luciferase reporter kit (Promega, Madison, WI, USA).

### Transmission electron microscopy (TEM) observations

According to a previously reported protocol [90], approximately 1-mm^3^ hippocampal pieces were fixed, stained en bloc, dehydrated and embedded in beam capsules. Then, 70- to 80-nm-thick sections of embedded tissue were collected onto grids and stained with uranyl acetate and lead citrate. TEM (JEOL, JEM-1011, Japan) was used for observation.

### Chromatin immunoprecipitation (ChIP) analysis

ChIP analysis was performed according to the manufacturer’s instructions (Millipore, CA, USA) to test the binding of NF-κB p65 to the miR-574-5p promoter. PROMO (http://alggen.lsi.upc.es/cgi-bin/promo_v3/promo/promoinit.cgi?dirDB=TF_8.3) was applied to find the NF-κB binding site within the promoter region. The primers for ChIP were as follows (amplicon size: 134 bp): 5′-AATCACATCCCACTCCCA-3′ (forward) and 5′-CCCTGCCTTCCATTACGA-3′ (reverse) for the binding site. Briefly, the hippocampal tissues were fixed with formaldehyde and washed twice with PBS containing protease inhibitors. The tissues were subsequently homogenized in PBS and centrifuged (2000 rpm, 6 min, 4 °C); then, the precipitates were resuspended in SDS lysis buffer and sonicated to obtain DNA lysate. The samples were centrifuged, and the supernatants were diluted with ChIP dilution buffer. The samples were subsequently precleared to reduce the nonspecific background and then incubated with primary antibody (anti-NF-κB) overnight. The antibody/histone complex was collected via the addition of Salmon Sperm DNA/Protein A Agarose Slurry, washed with different wash buffers, and eluted from the antibody; the protein and DNA cross-linking was reversed with NaCl. Finally, DNA was recovered via phenol/chloroform extraction and ethanol precipitation, and a PCR reaction and DNA agarose electrophoresis were conducted to obtain the final results.

### Morris water maze

Morris water maze studies with mice have principally been performed to measure hippocampal-dependent, spatially-based learning and memory. A circular water tank (diameter 100 cm for mice, and 75 cm tall) was filled with water rendered opaque by the addition of white, non-toxic paint. A circular platform with a diameter of 15 cm was placed 1 cm below the water surface in the center of a specific quadrant. If an animal failed to reach the platform within a fixed period of 60 s, it was gently guided to the location and was allowed to remain on it for 10 s. The animals were given training sessions using visible and invisible platforms for a period of 8 consecutive days (8 sessions in total). In the non-spatial water maze, the mice received 3 consecutive days of training to find the same platform elevated above the water surface. Invisible platform training was carried out for 5 continuous days (5 sessions), and each session consisted of 4 trials. For each trial, the mouse was released from the wall of the tank and allowed to search, find, and stand on the platform for 10 s within the 60-s trial period. For each training session, the sequence designed for the animals’ training was determined in a random manner that varied each day so that it differed in the separate sessions for each animal and was different for each individual animal. An EthoVision video tracking device (Noldus, Netherlands) was used to record the latency of reaching the target as a measure of task performance. The average latencies on each hidden platform were recorded and compared. Memory tests were conducted in a probe trial performed 24 h after the last training trial. A probe trial was performed in which the platform was removed from the tank, and the animals were allowed to swim for 60 s. The number of target annulus crossovers and the time spent in the target quadrant were measured.

### Electrophysiological recording

After the animal was sacrificed and the brain was dissected and kept in cold oxygenated artificial cerebrospinal fluid (ACSF), the slices (350–400 μm) were cut, moved to a holding chamber containing oxygenated ACSF at 34 °C for 0.5–1 h, and then kept in an incubator containing oxygenated ACSF at room temperature for recovery for more than 1.5 h. Before recording, each slice was transferred to a recording chamber continuously perfused with 95% O_2_ and 5% CO_2_-saturated ACSF at 32–34 °C. Field excitatory postsynaptic potential (fEPSP) recordings were made in response to stimulation of the perforant path in the DG region of the hippocampus at a frequency 0 of 0.05 Hz using an Axoclamp-2B patch-clamp amplifier (Molecular Devices, CA) in bridge mode. Hippocampal LTP was induced by theta burst stimulation (TBS) consisting of a series of 10 bursts of 5 pulses at 100 Hz.

### Immunoblot analysis

Proteins were extracted from hippocampal tissues and quantified according to our previously described method [93]. After SDS-polyacrylamide gel electrophoresis (SDS-PAGE), 50 μg of total protein was transferred to a nitrocellulose (NC) membrane and blocked with 3% bovine serum albumin (BSA). The membrane was incubated with rabbit β-actin (1:1000; Cell Signaling Technology, USA), RFP (1:1000; Abcam, UK), PSD-95, GluA1, GluA2, GluN1, GRIN2A, GRIN2B, BACE1, ADAM10, Nicastrin, APP, or GFP antibody (1:200; Bioss, China) at 4 °C overnight. The membrane was washed with PBS, incubated with IR Dye 800CW-conjugated secondary antibody (1:5000; LiCor Biosciences, USA), and subsequently detected with a LI-COR Odyssey Infrared Fluorescent System. The density of each band was quantified using Image-pro Express software, version 6.0 (Media Cybernetics, USA) and was normalized to the corresponding β-actin value to account for variations in loading.

### Enzyme-linked immunosorbent assay (ELISA) analysis

Levels of Aβ42 in hippocampal tissues and the concentrations of IL-1β and TNF-α in the lung, blood and hippocampal tissues were measured according to the manufacturer’s instructions (Westang Biotechnology Co., Shanghai, China). Approximately 0.04 g of tissue was homogenized with 400 μL 0.9% NaCl on ice. The homogenates were centrifuged for 20 min at 3000 rpm at 4 °C. The supernatant was then transferred to a new tube for analysis according to the ELISA reagent kit.

### Statistical analysis

The data are presented as the means ± S.E. Statistical significance was assessed using one-way analysis of variance (ANOVA) followed by LSD’s post hoc analysis for PM_2.5_ treatment and control/vehicle groups, and two-way ANOVA followed by Student’s *t*-test for rescue experiments. Differences in all tests were considered significant when *P* < 0.05.

## Additional files

Additional file 1: Figure S1. TEM observation of morphological alterations of synapse following PM_2.5_ exposure. **Figure S2.** BACE1 inhibition rescues morphological alterations of synapse. **Figure S3.** miR-574-5p overexpression recovers morphological alterations of synapse. **Figure S4.** A dual-luciferase analysis of NF-κB reporter activity was detected in HEK293T cells in response to PM_2.5_ stimulation. **Table S1.** Contents of PAHs, inorganic ions, carbon and elements in PM_2.5_ samples. (DOC 1544 kb)

## Abbreviations

ACSF: Artificial cerebrospinal fluid
AD: Alzheimer’s disease
AICD: APP intracellular domain
AMPA: α-amino-3-hydroxy-5-methyl-4-isoxazolepropionic acid
ANOVA: Analysis of variance
APP: Amyloid precursor protein
Aβ: Amyloid β
BACE1: β-site amyloid precursor protein cleaving enzyme 1
BSA: Bovine serum albumin
ChIP: Chromatin immunoprecipitation
DG: Dentate gyrus
ELISAs: Enzyme-linked immunosorbent assays
fEPSP: Field excitatory postsynaptic potential
GFP: Green fluorescent protein
LTP: Long-term potentiation
LV: Lentiviral vector
MiRNAs: MicroRNAs
NC: Nitrocellulose
NMDA: N-methyl-D-aspartate
PM: Particulate matter
PM_2.5_: Fine particles (PM ≤ 2.5 μm)
PSD-95: Postsynaptic density protein 95
qRT-PCR: Real-time quantitative reverse transcription PCR
RFP: Red fluorescent protein
SDS-PAGE: SDS-polyacrylamide gel electrophoresis
TBS: Theta burst stimulation
TEM: Transmission electron microscopy
UTR: Untranslated region
