# Supplementary material for: NF-κB-regulated microRNA-574-5p underlies synaptic and cognitive impairment in response to atmospheric PM2.5 aspiration
Source: Part Fibre Toxicol. 2017 Aug 29;14:34. doi: 10.1186/s12989-017-0215-3 (PMC5575838; doi:10.1186/s12989-017-0215-3)
Supplement: Supplementary file 1 — TEM observation of morphological alterations of synapse following PM2.5 exposure. Figure S2. BACE1 inhibition rescues morphological alterations of synapse. Figure S3. miR-574-5p overexpression recovers morphological alterations of synapse. Figure S4. A dual-luciferase analysis of NF-κB reporter activity was detected in HEK293T cells in response to PM2.5 stimulation. Table S1. Contents of PAHs, inorganic ions, carbon and elements in PM2.5 samples. (DOC 1544 kb) [file 12989_2017_215_MOESM1_ESM.doc]

**Additional file**

**NF-κB-regulated microRNA-574-5p underlies synaptic and cognitive impairment in response to atmospheric PM_2.5_ aspiration**

Authors: Tingting Ku, Ben Li, Rui Gao, Yingying Zhang, Wei Yan, Xiaotong Ji, Guangke Li, Nan Sang*

**Table of Contents**

Additional file 1: Figure S1 2

Additional file 1: Figure S2 3

Additional file 1: Figure S3 4

Additional file 1: Figure S4 5

Additional file 1: Table S1 6

Additional file 1: Figure S1


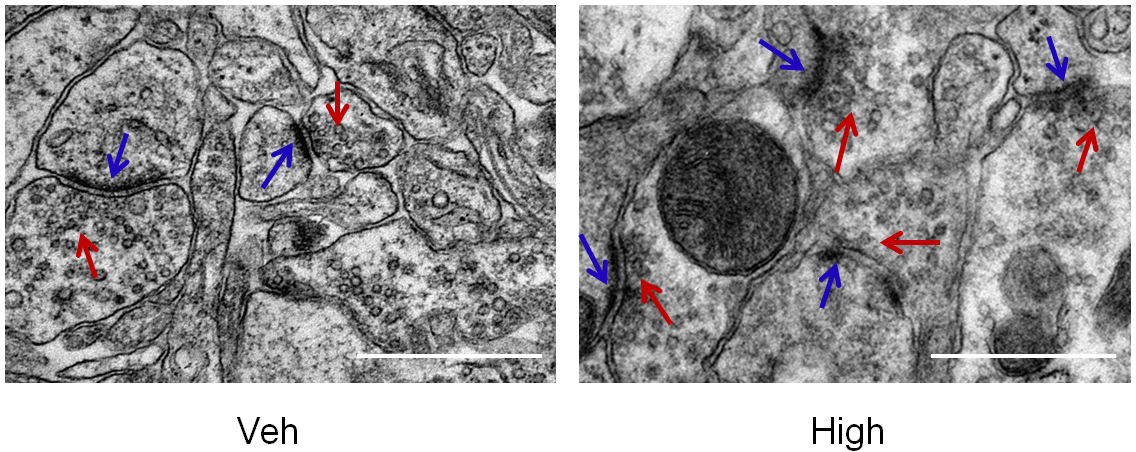


**Figure S1:** TEM observation of morphological alterations of synapse following PM_2.5_ exposure. Blue arrows show the postsynaptic density and red arrows show the synaptic vesicles in the hippocampus. Scale bars=500 nm. Veh=vehicle control; High=5 mg/kg PM_2.5_.

Additional file 1: Figure S2


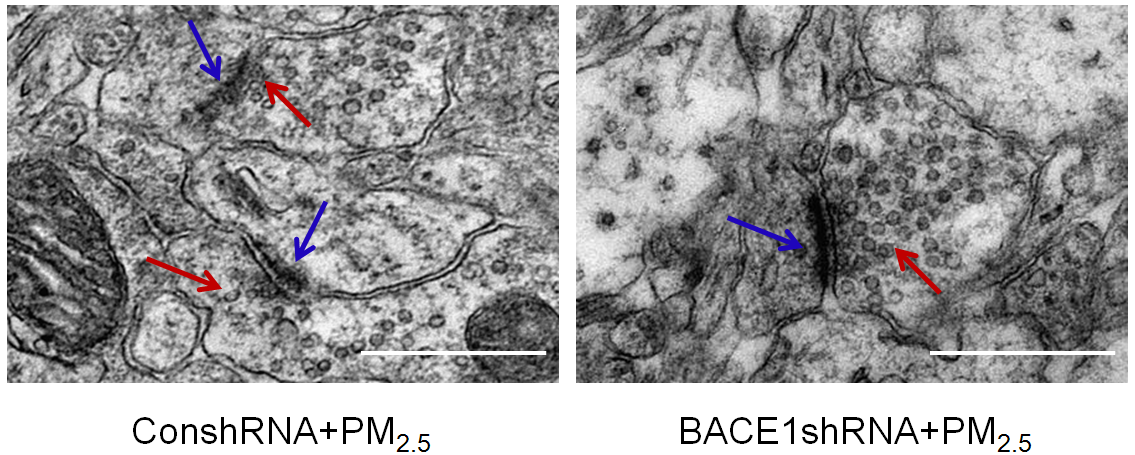


**Figure S2:** BACE1 inhibition rescues morphological alterations of synapse. Blue arrows show the postsynaptic density and red arrows show the synaptic vesicles in the hippocampus. Scale bars=500 nm.

Additional file 1: Figure S3


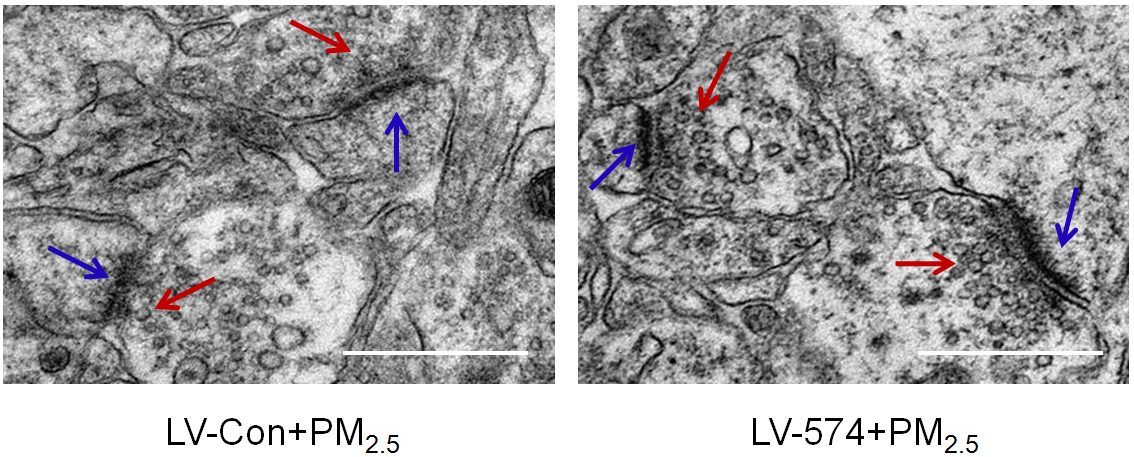


**Figure S3:** MiR-574-5p overexpression recovers morphological alterations of synapse. Blue arrows show the postsynaptic density and red arrows show the synaptic vesicles in the hippocampus. Scale bars=500 nm. LV-Con+PM_2.5_=LV-scramble control+PM_2.5_; LV-574+PM_2.5_=LV-miR-574-5p+PM_2.5_.

Additional file 1: Figure S4


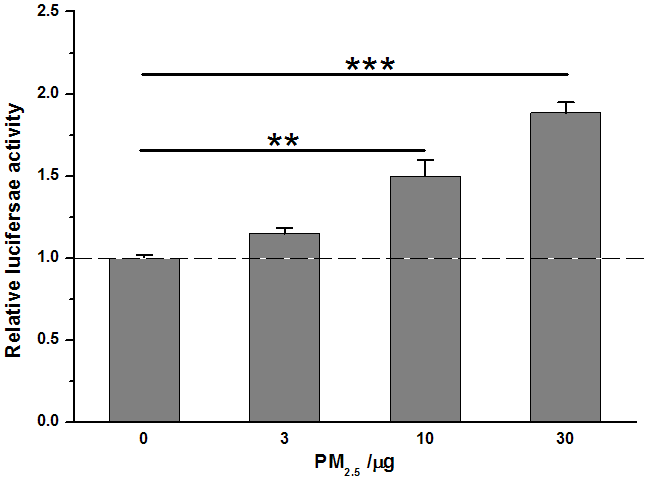


**Figure S4:** A dual-luciferase analysis of NF-κB reporter activity was detected in HEK293T cells in response to PM_2.5_ stimulation (*n=3*).

**Additional file 1: Table S1 Contents of PAHs, inorganic ions, carbon and elements in PM_2.5_ samples [1]**

| Polycyclic aromatic hydrocarbon(PAHs)  (ng/m^3^) | | Inorganic ions  (μg/m^3^) | | Carbon  (μg/m^3^) | | Elements  (ng/m^3^) | |
| --- | --- | --- | --- | --- | --- | --- | --- |
| Naphthalene (NA) | 0.222 | F^-^ | 0.030 | OC | 45.957 | Zr | 379.798 |
| Acenaphthylene (ACL) | 0.175 | Cl^-^ | 0.345 | EC | 15.476 | Al | 31313.131 |
| Acenaphthene (AC) | 0.451 | NO_3_^-^ | 0.088 | TC | 61.432 | Sr | 141.212 |
| Fluorene (FLU) | 1.672 | SO_4_^2-^ | 0.282 |  |  | Mg | 13737.374 |
| Benzo[g,h,i]pyrene (BPE) | 33.465 | Na^+^ | 3.409 |  |  | Ti | 1143.434 |
| Indeno[1,2,3-cd]pyrene (IPY) | 31.963 | NH_4_^+^ | 6.755 |  |  | Ca | 12585.859 |
| Dibenzo[a,h]anthracene (DBA) | 9.986 | K^+^ | 2.031 |  |  | Fe | 2262.626 |
| Benzo[b]fluoranthene (BbF) | 54.607 | Mg^2+^ | 0.240 |  |  | Ba | 1301.010 |
| Coronene (COR) | 10.189 | Ca^2+^ | 2.336 |  |  | Li | 80.000 |
| Phenanthrene (PHE) | 8.881 |  |  |  |  | Be | 2.020 |
| Anthracene (ANT) | 1.708 |  |  |  |  | Na | 14989.899 |
| Fluoranthene (FA) | 38.023 |  |  |  |  | P | 321.212 |
| Benzo[a]anthracene (BaA) | 38.438 |  |  |  |  | K | 8222.222 |
| Chrysene (CHR) | 39.493 |  |  |  |  | Sc | 6.061 |
| Pyrene (PYR) | 26.354 |  |  |  |  | V | 45.253 |
| Benzo[a]pyrene (BaP) | 32.636 |  |  |  |  | Cr | 41.616 |
| Benzo[e]pyrene (BeP) | 31.248 |  |  |  |  | Mn | 111.554 |
| Benzo[k]fluoranthene (BkF) | 16.456 |  |  |  |  | Co | 1.374 |
|  |  |  |  |  |  | Ni | 5.051 |
|  |  |  |  |  |  | Cu | 16.845 |
|  |  |  |  |  |  | Zn | 393.939 |
|  |  |  |  |  |  | Rb | 13.131 |
|  |  |  |  |  |  | Y | 10.101 |
|  |  |  |  |  |  | Mo | 2.828 |
|  |  |  |  |  |  | Cd | 2.020 |
|  |  |  |  |  |  | Sn | 7.273 |
|  |  |  |  |  |  | Sb | 25.253 |
|  |  |  |  |  |  | Cs | 3.232 |
|  |  |  |  |  |  | La | 21.818 |
|  |  |  |  |  |  | Ce | 292.929 |
|  |  |  |  |  |  | Sm | 1.919 |
|  |  |  |  |  |  | W | 5.051 |
|  |  |  |  |  |  | Tl | 1.212 |
|  |  |  |  |  |  | Pb | 127.071 |
|  |  |  |  |  |  | Bi | 2.828 |
|  |  |  |  |  |  | Th | 8.687 |
|  |  |  |  |  |  | U | 2.828 |

Reference:

[1] Chen M, Li B, Sang N. Particulate matter (PM_2.5_) exposure season-dependently induces neuronal apoptosis and synaptic injuries. J Environ Sci. 2017;54:336-45.
